# Deep Sequencing Identifies Ethnicity-Specific Bacterial Signatures in the Oral Microbiome

**DOI:** 10.1371/journal.pone.0077287

**Published:** 2013-10-23

**Authors:** Matthew R. Mason, Haikady N. Nagaraja, Terry Camerlengo, Vinayak Joshi, Purnima S. Kumar

**Affiliations:** 1 Division of Oral Biology, College of Dentistry, The Ohio State University, Columbus, Ohio, United States of America; 2 Division of Biostatistics, College of Public Health, The Ohio State University, Columbus, Ohio, United States of America; 3 Comprehensive Cancer Center, The Ohio State University, Columbus, Ohio, United States of America; 4 Department of Periodontics, Maratha Mandal’s NGH Institute of Dental Sciences and Research Centre, Belgaum, India; 5 Division of Periodontology, College of Dentistry, The Ohio State University, Columbus, Ohio, United States of America; University of Kansas Medical Center, United States of America

## Abstract

Oral infections have a strong ethnic predilection; suggesting that ethnicity is a critical determinant of oral microbial colonization. Dental plaque and saliva samples from 192 subjects belonging to four major ethnicities in the United States were analyzed using terminal restriction fragment length polymorphism (t-RFLP) and 16S pyrosequencing. Ethnicity-specific clustering of microbial communities was apparent in saliva and subgingival biofilms, and a machine-learning classifier was capable of identifying an individual’s ethnicity from subgingival microbial signatures. The classifier identified African Americans with a 100% sensitivity and 74% specificity and Caucasians with a 50% sensitivity and 91% specificity. The data demonstrates a significant association between ethnic affiliation and the composition of the oral microbiome; to the extent that these microbial signatures appear to be capable of discriminating between ethnicities.

## Introduction

Personalized medicine is based on the paradigm that factors affecting disease susceptibility are unique to each individual and are significantly influenced by the genotype of the host, of which, gender, race/ethnicity and genetics are critical determinants. This is true not only of diseases like diabetes, stroke and hypertension, but also of acute and chronic microbial infections. For example, host genotype significantly affects susceptibility to cholera [1], pneumonia [2] and cystic fibrosis [3]. While this could imply a genetic inability to mount an effective immune response to infections, several lines of evidence have also emerged, showing that the host genotype plays an important role in bacterial colonization [4].

Oral bacteria colonize the oral cavity a few minutes after birth and form stable ecosystems in several niches within this ecosystem. Two of the most common diseases to affect humans, caries and periodontal diseases, result from perturbations of these indigenous bacterial communities. Additionally, evidence is emerging to show that oral microbial communities play a critical role in the pathogenesis of oral cancer [5], [6]. It has been established that susceptibility to these diseases varies among ethnicities after controlling for socioeconomic, dietary, and other environmental factors [7], suggesting that indigenous oral microbial communities differ between ethnicities and that these differences may underlie the differential disease susceptibilities. Therefore, we investigated if variations in the composition of health-compatible oral biofilms can be attributable to an individual’s ethnic affiliation.

## Results

### Clinical and Demographic Features

We compared the oral microbial communities of 192 people belonging to four ethnic affiliations: non-Hispanic blacks (AA), non-Hispanic whites (CA), Chinese (CH), and Latinos (LA). These ethnicities were selected since they represent four major races/ethnic groups residing in the United States. All subjects reported both parents and both sets of grandparents to be of the same ethnicity; Chinese and Latino subjects were either immigrants from China and Taiwan, or Central America and Puerto Rico respectively, or first generation residents. All subjects were free of systemic diseases, active caries, and periodontal diseases (periodontitis and gingivitis).

### Comparison of Microbial Signatures Using t-RFLP

We used terminal restriction fragment length polymorphism (t-RFLP), to compare the signatures of the salivary, supragingival and subgingival microbiomes between the four ethnic groups. These environments represent three distinct microbial niches within the oral ecosystem. Supragingival plaque forms on the tooth surface that is exposed to mechanical and frictional forces and is influenced by the lifestyle of the individual (for example, food and oral hygiene habits) and hence, represents a biofilm where the effects of the environment supercede the effects of the host genotype. The subgingival biofilm on the other hand, represents a community that is influenced to a large extent by genetically controlled host-associated factors (for example tooth morphology, epithelial barrier function, and innate immune responses). Saliva represents a fluid environment in communication with all oral habitats, and hence shares microbial fingerprints with both supragingival and subgingival ecosystems.

**Figure 1:**
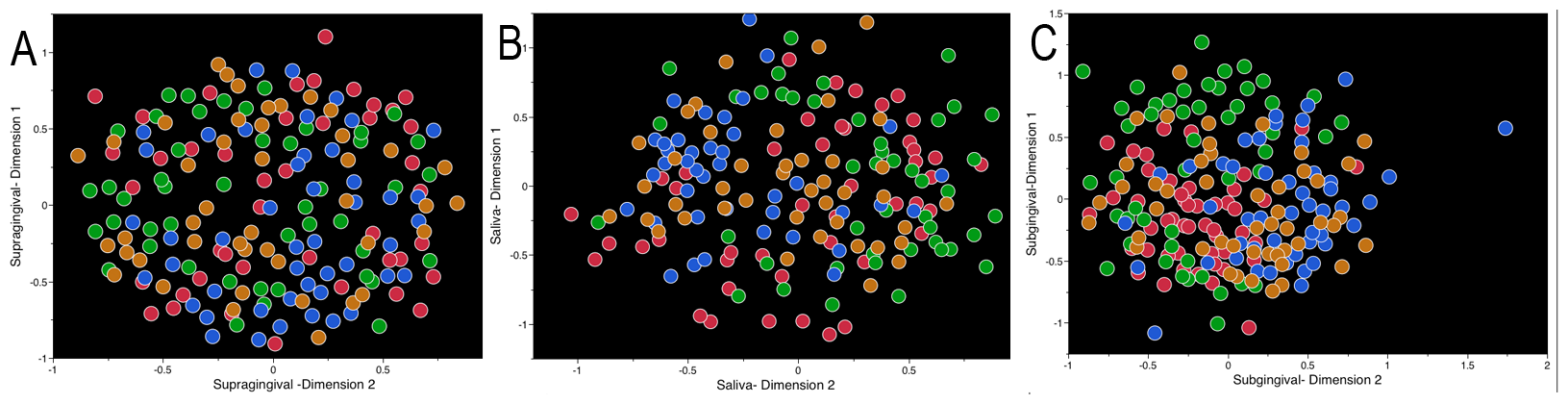
Non-metric multidimensional scaling (NMDS) of t-RFLP peak areas. Subgingival plaque is shown in Figure 1A, saliva in 1B, and supragingival plaque in 1C. Non-Hispanic blacks are indicated by red, non-Hispanic whites are in green, Chinese are in blue, and Latinos are indicated by orange. Significant ethnicity-based clustering was seen in subgingival and saliva samples (Subgingival stress value = 0.09, Saliva stress value = 0.11, Supragingival stress value = 0.12).

16S rRNA genes were amplified using broad-range fluorescent-labeled primers and subsequently digested using restriction enzymes, generating terminal fragments of varying lengths based on species-specific differences in the location of the restriction sites. Thus, the total number of peaks represented the number of unique species present in the community and the area of each peak represented the abundance of each species. Non-metric Multi-Dimensional Scaling (PROXSCAL NMDS) of the Bray Curtis Similarity Index [8] was used to examine the strength of clustering of the microbial communities from the three oral niches. We found that the subgingival community demonstrated the strongest ethnicity-specific clustering, followed by the salivary samples, and no clustering in the supragingival communities (Figure 1a–c), suggesting that ethnicity exerts a selection pressure by influencing host-associated bacterial colonization factors.

### Comparison of Microbial Signatures using 16S Pyrotag Sequencing

**Figure 2:**
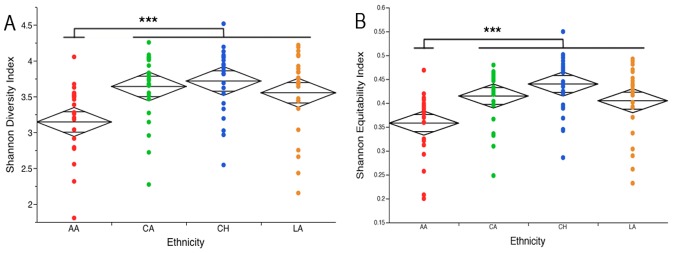
Shannon diversity and equitability indices by ethnicity. Diversity is shown in Figure 2A and equitability in Figure 2B. Non-Hispanic blacks demonstrated significantly lower bacterial diversity (***p<0.001, ANOVA) and equitability (***p<0.001, ANOVA) compared to the other ethnicities.

Since the t-RFLP demonstrated early evidence of clustering, we characterized the bacterial lineages of the subgingival microbiome in 100 randomly selected individuals, 25 from each of the four ethnic groups, using multiplexed 16S pyrotag sequencing. For each sample, variable regions V1–V3 and V7–V9 of the bacterial 16S ribosomal RNA (rRNA) gene were sequenced and combined to create a composite dataset. A total of 633,601 high-quality, chimera-depleted, classifiable sequences were obtained. These sequences represented 398 species-level operational taxonomic units (s-OTUs) with an average of 149±34 s-OTUs detected in each individual. S-OTU data was used to compute Shannon Diversity and Equitability indices. The Shannon index incorporates both the number of s-OTUs (richness) and relative abundance of each s-OTU (evenness) into a single value. While a Diversity Index of zero represents a mono-species community, a higher value may result either from the presence of several species or from equitable distribution of a few species. Thus, the Equitability index serves to characterize the relative contributions of species richness and evenness to the Diversity index. African Americans had lower Diversity(p = 0.0006, ANOVA) (Figure 2a) and Equitability (p = 0.0002, ANOVA) (Figure 2b) indices when compared to the other three ethnic groups. This indicates that African Americans have fewer subgingival species and that a few of these species are numerically dominant members of the community when compared to the other ethnicities.

### Existence of a Core Microbiome across Individuals and within Ethnicities

**Figure 3:**
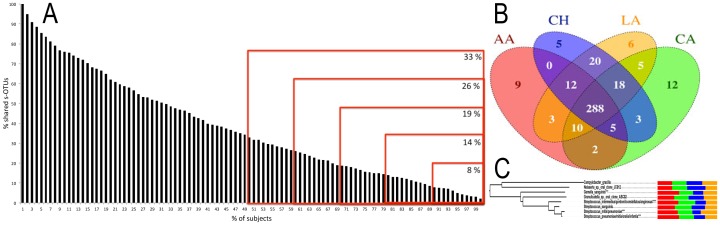
Shared species at subject-level (Figure 3A) and ethnicity-level (Figure 3B). 2% of s-OTUs were shared by all individuals, 8% of s-OTUs were shared by 90% of individuals and over a third of the s-OTUs were shared by 50% of the subjects, supporting the existence of a core microbiome at both levels. Figure 3C shows relative abundances of species shared by all individuals. Abundances of 4 shared species were different between ethnicities (p<0.001, ANOVA on transformed variable).

**Figure 4:**
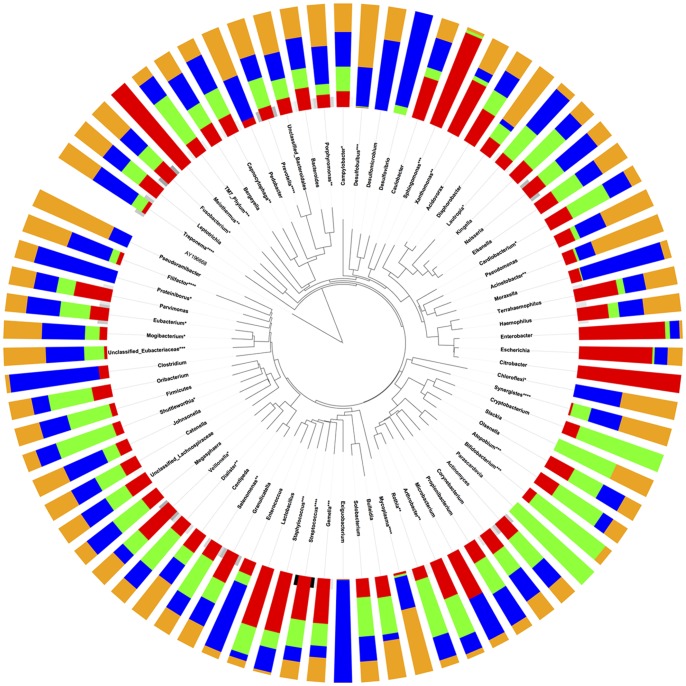
Relative abundances of 77 genus-level OTUs(g-OTUs). Figure was generated using ITOL. Non-Hispanic blacks are indicated by red, non-Hispanic whites are indicated by green, Chinese are indicated by blue, and Latinos are indicated by orange. The abundances of four s-OTUs and several g-OTUs were significantly different between ethnicities (*p<0.05, **p<0.01, ***p<0.001, ****p<0.0001, ANOVA on transformed variable).

The Human Microbiome Project has highlighted the importance of identifying a ‘core microbiome’ that is common to all healthy individuals, in order to understand susceptibility to disease. We found eight s-OTUs (2%) that were present in all 100 individuals (Figure 3a). Moreover, 8% of the 398 s-OTUs were detected in 90% of individuals and over a third of the s-OTUs were shared by half of the subjects (Figure 3a). These findings support the existence of a ‘core microbiome’ within the subgingival habitat. However, we also found the existence of s-OTUs unique to each ethnicity (Figure 3b) indicating a possible ethnicity-based selection in the composition of the subgingival microbial community. Furthermore, half of the eight s-OTUs present in all subjects showed significant differences in abundances between ethnicities (Figure 3c) lending further support to the fact that ethnicity plays a role in determining the composition of the subgingival microbiome. Analysis of the datasets at the genus level further served to confirm this finding, since 33 of the 77 genera demonstrated significant differences in abundance between the ethnic groups (p<0.05, ANOVA) (Figure 4). This suggests that distinct bacterial lineages contribute to the composition of the subgingival communities in different ethnicities.

### Microbial Signature Predicts an Individual’s Ethnicity

**Figure 5:**
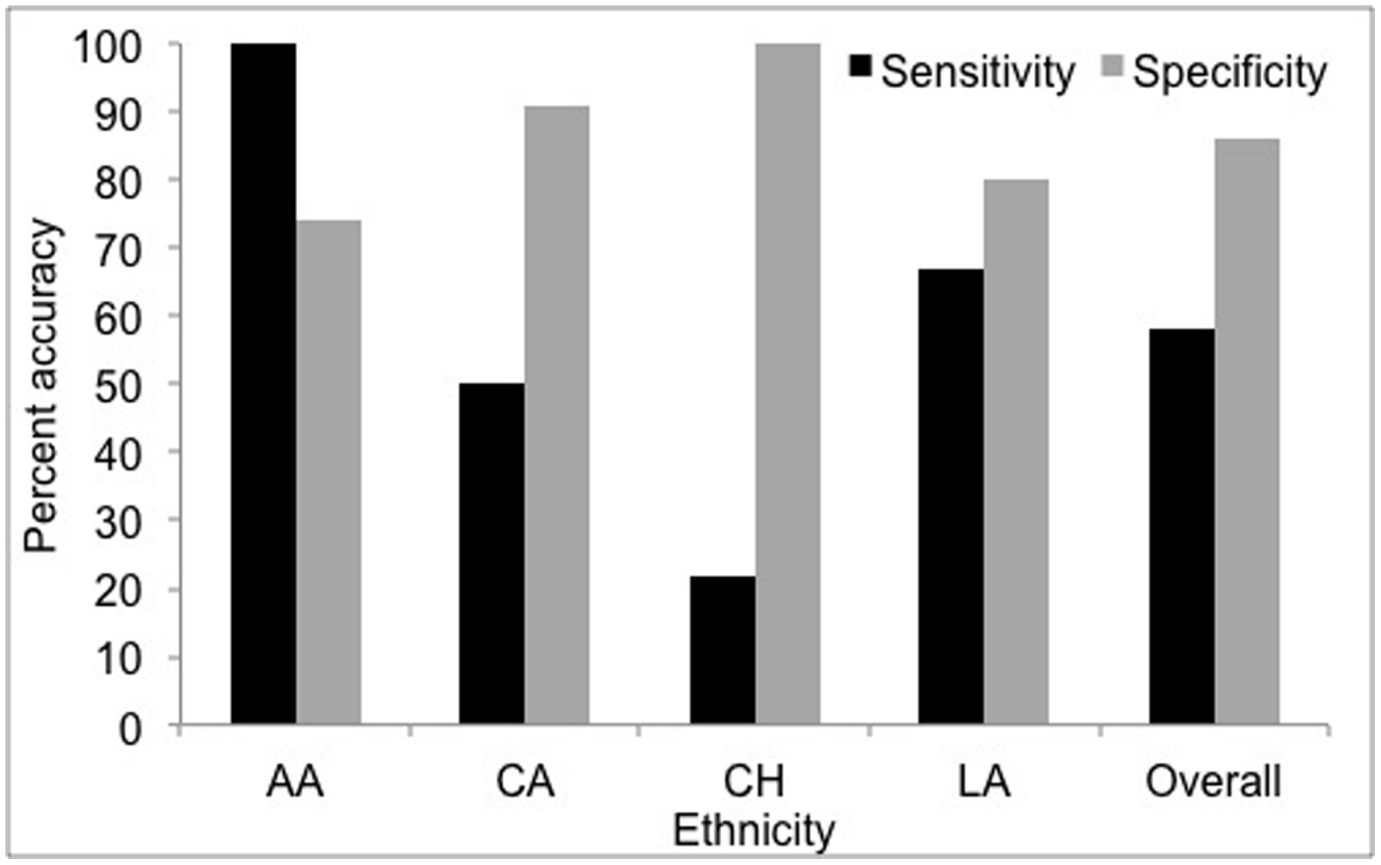
Accuracy of discriminating between ethnicity using the subgingival microbial signature. Non-Hispanic Blacks (AA) demonstrated the greatest accuracy, with 100% sensitivity and 74% specificity, followed by Latinos(LA) and Caucasians (CA).

We found that the subgingival microbial fingerprint can successfully discriminate between the four ethnicities. To do this, a Random Forest machine-learning classifier was trained to develop an educated classification algorithm using subgingival microbial signatures, which was then applied to a test dataset to examine the accuracy, sensitivity and specificity of the prediction. The subgingival microbial community was able to predict an individual’s ethnicity with a 62% accuracy, 58% sensitivity and 86% specificity (Figure 5). The classifier was able to predict African Americans with a 100% sensitivity and 74% specificity, followed by Latinos (67% and 80%) and Caucasians (50% and 91%) (Figure 5). This is interesting because although African Americans and Caucasians have shared similar environmental factors including food, nutrition, and lifestyle over several generations, (unlike Chinese and Latino subjects who were either immigrants or first generation residents), they demonstrated distinct microbial communities. This suggests that the host genotype influences the microbial community to a greater extent than shared environment; “nature” appears to win over “nurture” in shaping this community.

**Figure 6:**
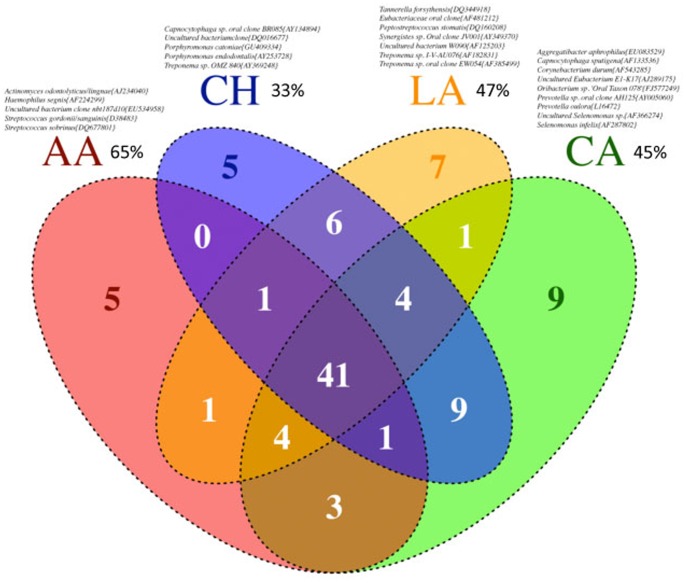
Venn diagram of ethnicity specific s-OTUs. These OTUs were identified based on presence/absence in each individual. The likelihood of discriminating between ethnicities using presence/absence of these s-OTUs is shown in parenthesis. The mere presence of these ethnicity-specific consortia was capable of identifying an individual’s ethnicity better than chance.

We then investigated if the mere presence a consortium of selected microbial species could be used as surrogates to predict an individual’s ethnicity. To do this, we identified species that were present in at least 80% of the subjects within each ethnicity (Figure 6). We choose 80% as our minimum detection threshold because this level included species with varying abundances. Next, we estimated the likelihood that these microbial consortia will predict an individual’s ethnicity. This method demonstrated a prediction likelihood of 65% for African Americans, 45% for Caucasians, 33% for Chinese, and 47% for Latinos. Thus, it is possible that these bacterial consortia are capable of discriminating between ethnicities, and it is important to validate this panel in a larger population.

## Discussion

A fundamental tenet in microbial ecology is that “Everything is everywhere, but the environment selects” [9]. Our data demonstrates that ethnicity exerts a selection pressure on the oral microbiome, and that this selection pressure is genetic rather than environmental, since the two ethnicities that shared a common food, nutritional and lifestyle heritage (Caucasians and African Americans) demonstrated significant microbial divergence. It is known that tooth and root morphologies vary according to ethnic affiliation [10], [11], as do innate immune responses to infectious agents, for example, Toll-like receptor-4, mannose binding lectin and heat-shock proteins [12], [13] and it is possible that ethnicity plays a role in bacterial selection by defining the environment for bacterial colonization.

The concept that the host genotype chooses what will survive and thrive is particularly important in assessing susceptibility and in developing targeted therapies to combat polymicrobial infections. For example, pathogens belonging to the genera *Filifactor, Staphylococcus, Mycoplasma,* and *Treponema* were found in significantly higher levels in Chinese and Latinos, and it is possible that their presence in health may contribute to the increased disease susceptibility that has been observed in these cohorts [14], [15]. Successful treatment of biofilm-associated diseases requires re-establishing healthy oral biofilms, and current treatment protocols assume that the composition of health-compatible biofilms are similar among all populations. The data presented here suggest that the health-compatible communities consist of significantly different types of species as well as different ratios of common species between different ethnicities and therefore, microbial replacement therapy has to be tailored to cohorts rather than be universally applied.

In summary, the work presented here demonstrates the existence of ethnicity-specific subgingival microbiomes that are characterized by differing bacterial lineages and varying diversities. It is possible that these health-associated ethnicity-specific microbial communities may predispose individuals to future disease and warrants further examination.

## Materials and Methods

### Study Population

Approval for this study was obtained from the Office of Responsible Research Practices at The Ohio State University (Protocol Number: 2008H0122). Periodontally and dentally healthy individuals over 18 years of age were recruited from those responding to recruiting campaigns. All subjects interested in the study were emailed a screening questionnaire. This electronic interview served to exclude subjects who were below 18 years of age and satisfy the exclusion criteria listed. Subjects who reported diabetes, HIV, pregnancy, immunosuppressant medications, bisphosphonates or steroids, current smoking history, current orthodontic therapy, antibiotic therapy or professional cleaning within the previous 3 months, as well as those who required antibiotic coverage before dental treatment, and those who did not meet the ethnicity requirements were excluded from this study. A total of 192 subjects successfully completed the study. Each ethnic group, including African American, Caucasian, Chinese, and Latino, was represented by 48 subjects.

### Initial Clinical Screening

Qualifying subjects participated in a periodontal examination to ensure that they satisfied the clinical criteria for inclusion into the study. All subjects were examined by calibrated periodontists. Gingival and plaque indices were recorded throughout the mouth using a PCP-UNC 15 probe. Subjects with at least 20 natural non-carious teeth, ≤3 mm probing pocket depths at all sites (indicative of healthy gums), average pre-brushing plaque score of ≤ 1.9 (Quigley-Hein modification of the Turesky Plaque Index TPI) [16] and a Loe and Silness gingival index (GI) [17] of ≤1 were selected using this clinical examination.

### Informed Consent and Inclusion into Study

Each subject who qualified for the study was explained the purpose and procedures of the research and written informed consent obtained.

### Sample Collection

Saliva was collected by expectorating into a sterile 1.5 mL tube using a methodology as previously described [18]. Briefly, subjects will be asked to collect saliva in their mouth for 3 minutes and then continuously drool into a tube for 3 minutes. This method will allow us to collect unstimulated saliva that will contain significantly greater numbers of bacteria than simply spitting into a tube. Supragingival plaque was collected from interproximal sites using scalers. Following supragingival plaque removal, the area was isolated and subgingival plaque was collected by inserting endodontic paperpoints (Caulk Dentsply) into the interproximal gingival sulci of 10 randomly selected teeth. All the paper point and scaler samples were pooled.

### DNA Isolation

A previously described methodology for DNA isolation was used [19]. For saliva samples, 50 µl of saliva was added to 200 µl of phosphate buffered saline (PBS) before preceding with isolation using a Qiagen MiniAmp kit (Valencia, CA) according to manufacturer’s instructions. For plaque samples, bacteria were removed from the paper points by adding 200 µl of phosphate buffered saline (PBS) and vortexing for 1 minute. The paper points were then removed, and DNA isolated using a Qiagen MiniAmp kit (Valencia, CA) according to the manufacturer’s instructions.

### t-RFLP Analysis

Bacterial 16S rRNA genes were amplified using 22 cycles of PCR with fluorescent- labeled broad range bacterial primers A18-FAM (5′- TT TGA TCC TGG CTC AG–FAM-3′) and 317-HEX (5′- FAM-AAG GAG GTG ATC CAG GC -3′) (Applied Biosystems, Foster City, CA). The cycling conditions have previously been described [20]. The amplicons were purified using a Qiaquick kit (Qiagen, Valencia, CA). Restriction digestion was carried out with 10 µl of standardized, purified PCR product and 10 U of *Msp I* in a total volume of 20 µl at 37°C for three hours. 10 µl of the digestion product was purified using AMPure beads (Agencourt Bioscience Corporation, Beverly, MA) according to the manufacturer’s protocol and eluted in 50 µl water. 5 µl of the purified product was denatured with 10 µl of deionized formamide and mixed with 0.2 µl GeneScan 1200 LIZ size standard (Applied Biosystems, Foster City, CA). Fragment lengths were determined on an AB 3730 DNA Analyzer in GeneScan mode. The number of peaks as well as the height and area of each peak; reflecting the sizes and intensities of the terminal fragments were determined using the GeneMapper 4.0 Software.

Peak areas were standardized by converting the raw values to a proportion of the total area as previously described [21]. Peaks representing less than 1% of the total area were assigned a value of zero and the percentages of the remaining peaks recalculated. A variance stabilizing transformation was used to create normal distribution of the data [22]. The proportion (p) of each peak in the community of each subject was expressed as X = sin ^−1^(√p) and were used for nonmetric multidimensional scaling (NMDS) computed within SPSS (IBM, Armonk, NY). Visualization was carried out with JMP (SAS Institute Inc., Cary, NC).

### Pyrosequencing

Multiplexed bacterial tag-encoded FLX amplicon pyrosequencing (bTEFAP) was performed using the Titanium platform (Roche Applied Science, Indianapolis, IN) as previously described [23] in a commercial facility (Research and Testing Laboratories, Lubbock, TX). Briefly, a single step PCR with broad-range universal primers and 22 cycles of amplification was used to amplify the 16S rRNA genes as well as to introduce adaptor sequences and sample-specific bar-code oligonucleotide tags into the DNA. Two regions of the 16S rRNA genes were sequenced: V1–V3 and V7–V9. The primers used for sequencing have been previously described (Kumar et al, PlosONE). Adaptor sequences were trimmed from raw data with 98% or more of bases demonstrating a quality control of 30 and sequences binned into individual sample collections based on bar-code sequence tags, which were then trimmed. The resulting files were denoised with Pyronoise [24] and depleted of chimeras using B2C2 (http://www.researchandtesting.com/B2C2.html). Sequences <300 bp were discarded and the rest were clustered into species-level operational taxonomic units (s-OTUs) at 96% sequence similarity and assigned a taxonomic identity by alignment to locally hosted version of the Greengenes database [25] using the Blastn algorithm. Phylogenetic trees were generated by MacVector and visualized using iTOL [26]. Community diversity metrics were computed as previously described [27].

### Statistical Analysis

Shannon diversity index was computed using s-OTU data [28]. A variance stabilizing transformation was used to create normal distribution of the data [22]. The proportion (p) of each s-OTU in the community of each subject was expressed as X = sin ^−1^(√p) and ANOVA and 2-sample t-tests were used to compare the means of this transformed variable X across groups. Species and genera shared by ethnic groups were identified used to compute both the core microbiome as well as ethnicity-specific microbiomes. Species present in >80% of each ethnic group were considered for analysis. Discriminant analysis of each individual’s microbial community was performed using a trained random forest machine learning algorithm carried out with Statistica (StatSoft Inc., Tulsa, OK). To predict the likelihood that an individual was of a certain ethnicity given their microbial signature we calculated the number of subjects in an ethnic group that contained >80% of their respective ethnicity-specific microbiome species divided by the total number of subjects from different ethnicities who also contained >80% of the numerator’s ethnicity-specific microbiome species. Statistical analysis was carried out with JMP (SAS Institute Inc., Cary, NC) and graphics created using R (http://www.r-project.org/).
