# Correction: Deep Sequencing Identifies Ethnicity-Specific Bacterial Signatures in the Oral Microbiome

**DOI:** 10.1371/journal.pone.0099933

**Published:** 2014-06-04

**Authors:** Matthew R. Mason, Haikady N. Nagaraja, Terry Camerlengo, Vinayak Joshi, Purnima S. Kumar

The authors would like to supply additional information in relation to the study.

The characteristics of the sample population included in the study are outlined in the table supplied. The methods pertaining to this are as follows:

Informed consent and inclusion into study: Each subject who qualified for the study was explained the purpose and procedures of the research and written informed consent obtained. Questionnaires were used to assess demographics, oral hygiene and diet. Oral hygiene habits were assessed by frequency of brushing, flossing, smoking history and visits to the dentist, while food habits were assessed by routine exposures to one of the four diet patterns: (i) Standard American (diet rich in red meat, bologna, sausages, hotdogs and similar foods, refined grains (for example, bread), pasta and similar dishes, and desserts). (ii) Hispanic and Latino (diet rich in rice, beans, corn (as flour, e.g. tortilla, or fresh), avocado, olives, fruits, sweet breads, red meats), (iii) Asian (diet rich in rice, noodles, millet, fresh and salted vegetables, seafood, poultry, eggs, legumes) or (iv) not fitting into any of the above categories.

The legend for Figure 1 should be revised to read as below:

**Figure 1:**
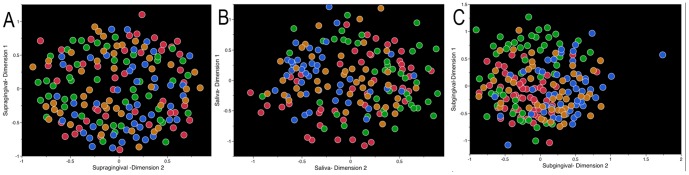
Non-metric multidimensional scaling (NMDS) of t-RFLP peak areas. Supragingival plaque is shown in Figure 1A, saliva in 1B, and subgingival plaque in 1C. Non-Hispanic blacks are indicated by red, non-Hispanic whites are in green, Chinese are in blue, and Latinos are indicated by orange. Significant ethnicity-based clustering was seen in subgingival and saliva samples (Subgingival stress value = 0.09, Saliva stress value = 0.11, Supragingival stress value = 0.12)

## Supporting Information

Table S1 **Ethnicity Table**
(JPG)
