# Supplementary figures and images for: Correction: Deep Sequencing Identifies Ethnicity-Specific Bacterial Signatures in the Oral Microbiome
Source: PLoS One. 2014 Jun 4;9(6):e99933. doi: 10.1371/journal.pone.0099933 (PMC4045986; doi:10.1371/journal.pone.0099933)

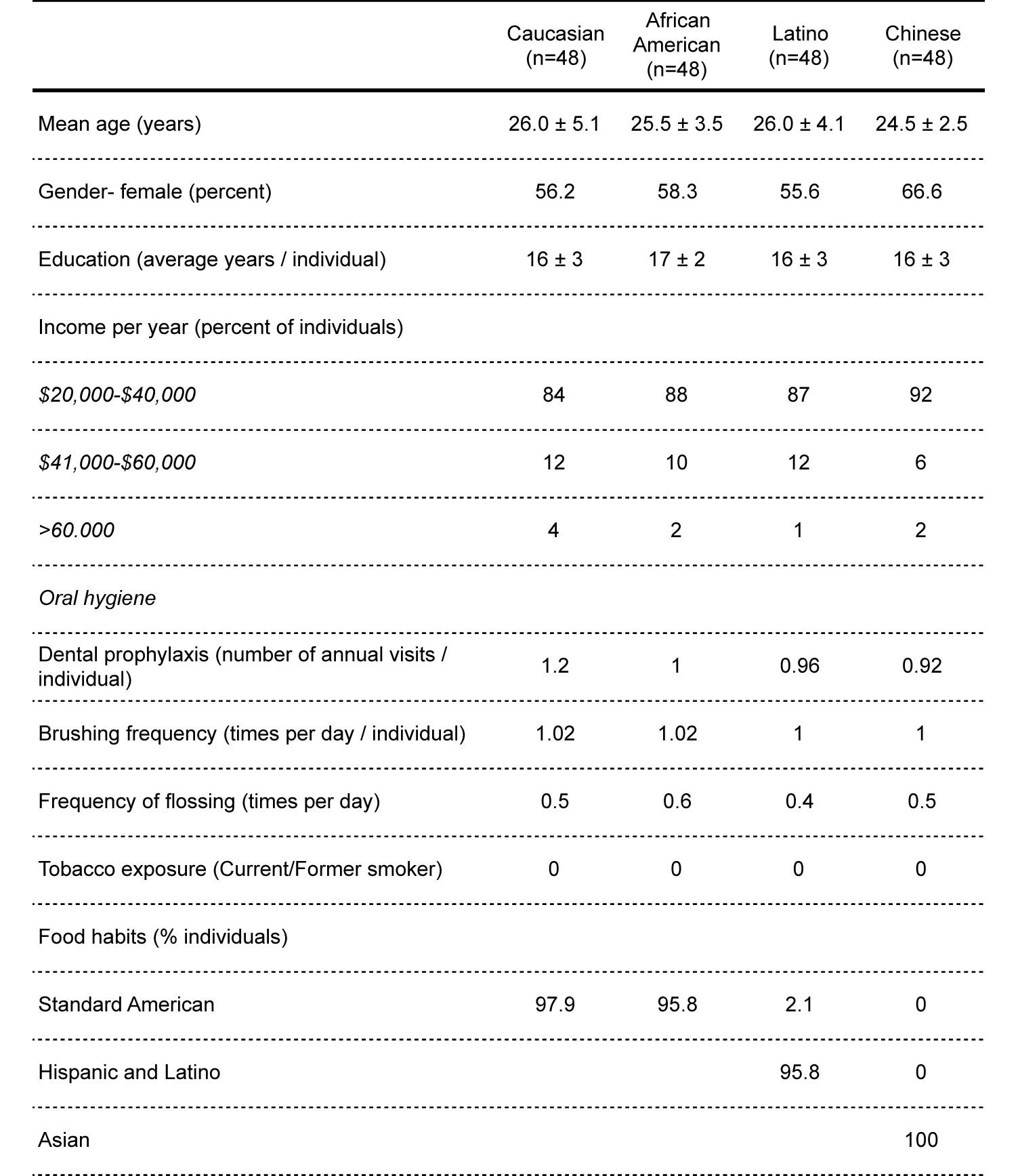

Supplement: Table S1 — Ethnicity Table (JPG) [file pone.0099933.s001.jpg]
